# M channels and stress response

**DOI:** 10.18632/oncotarget.17110

**Published:** 2017-04-12

**Authors:** Sheng Wang, Fang Yuan, De-Pei Li

**Affiliations:** Department of Critical Care, The Unversity of Texas, MD Anderson Cancer Center, Houston, TX, USA

**Keywords:** stress, potassium channels, neuroendocrine, neuroscience

Stress responses to environmental or psychological challenges such as traumatic events are mediated by the autonomic nervous system and neuroendocrine system. Rapid responses of these 2 systems are essential for the individual survival. Recent studies have attempted to elucidate the neuroplasticity in the hypothalamus in response to stress stimuli. Both acute restraint stress and chronic unpredictable stress reduce presynaptic GABA release to neuroendocrine neurons in the paraventricular nucleus of the hypothalamus (PVH). Furthermore, both acute and chronic stress diminish GABAergic inhibition in the PVH due to a depolarizing shift of GABA reversal potential in the PVN neurons [1, 2]. The alteration of chloride homeostasis, which determines GABA reversal potential, is caused by a downregulation of the cation-chloride co-transporter K^+^-Cl^–^-Cl^–^ (KCC2) during acute stress [1], but by an upregulation of cation-chloride co-transporter Na^+^-K^+^-Cl^–^-Cl^–^ (NKCC1) in response to chronic stress [2]. In our recent study, Zhou et al found that acute restraint stress suppresses M-current to increase the activity of corticotropin-releasing hormone (CRH)-expressing neurons in the PVH and hyperactivity of the hypothalamic–pituitary–adrenal (HPA) axis [3]. This study provides evidence that, in addition to altering synaptic inputs, acute stress affects neuroendocrine function through regulating ion channel activity.

The non-inactivating and voltage-dependent M-current stabilizes cell membrane potential because the M-channels are open at rest membrane potential and open even more during depolarization [4]. Thus, M-channels play a key role in raising the threshold for action potential firing. The M-channels are composed of 5 members of the Kv7 family of K^+^ channels, which are encoded by KCNQ genes [5]. The neuronal M-channel is predominantly carried by heterotetrameric Kv7.2 and Kv7.3 subunits; and other combinations, including Kv7.4 and Kv7.5 subunits add functional and pharmacological diversity. Mutations in Kv7 subunits are associated with human disorders such as deafness (Kv7.4), long-QT syndrome (Kv7.1), and epilepsy (Kv7.2 and Kv7.3), suggesting that Kv7 channels are crucially involved in a wide variety of physiological functions [5]. In addition, the activation of Kv7 channels in rats prevents impairments of hippocampal long-term potentiation and spatial memory retrieval caused by acute stress. The study by Zhou et al provides evidence that acute stress alters Kv7 function in the control of HPA axis activity. Furthermore, increased activity of AMP-activated protein kinase (AMPK) mediates the reduction of Kv7.3 subunit function in the CRH neurons during acute stress [3]. AMPK activation reduces membrane expression of Kv7.1 through promoting endocytosis and degradation in lysosomes via a Nedd2-4-dependent mechanism [6]. Thus, studies are needed to elucidate whether the reduced expression of Kv7.3 subunits is due to Nedd2-4-mediated endocytosis and degradation in lysosomes, which promote AMPK activation. Although the study by Zhou et al indicates that Nedd2-4-dependent endocytosis and degradation are likely involved in acute stress-induced decrease in Kv7.3 subunit in the PVH [3], one should not ignore the role of epigenetic regulation of KCNQ gene expression in response to acute stress [7].

It should be noted that the stress levels and the length of stress stimuli might affect M-channel function because it has been shown that acute restraint stress decreases KCC2 expression levels while chronic unpredictable stress induces a transient decrease of KCC2 and a long-lasting upregulation of NKCC1 [2]. Thus, it is reasonable to predict that acute or chronic stress may differently affect ion channel functions. In addition, it is worthwhile to get more information about the impact of acute stress on the function of ion channels such as A-type transient K^+^ channels, Ca^2+^-activated K^+^ channels, ATP-dependent K^+^ channels, sodium channels, and calcium channels. It is well established that acute or chronic stress may lead to major depression and posttraumatic stress disorder (PTSD), which are associated with hyperactivity of the HPA axis [8]. Because M channels are crucial in the regulation of the HPA axis activity, treating major depression and PTSD through targeting M channels to suppress HPA axis activity is novel therapeutic strategy.

## References

[1] Hewitt SA (2009). Nat Neurosci.

[2] Gao Y (2017). Neuroendocrinology.

[3] Zhou JJ (2017). Neuropharmacology.

[4] Brown DA (1980). Nature.

[5] Brown BS (2000). Prog Biophys Mol Biol.

[6] Bhalla V (2006). J Biol Chem.

[7] Stankiewicz AM (2013). Brain Res Bull.

[8] Gold PW (2015). Mol Psychiatry.

